# Could Hepcidin Be a Phenotype-Related Biomarker in Patients with Stable Bronchiectasis?

**DOI:** 10.3390/life16091439

**Published:** 2026-08-29

**Authors:** Gulcin Yilmaz Gunes, Hikmet Çoban, Fuat Erel, Merve Akış Yılmaz, Nurhan Sarioglu, Mustafa Colak, Merve Yumrukuz Senel, Ayşe Dilvin Mansır Elmalı

**Affiliations:** 1 Department of Pulmonology, Bandırma Training and Research Hospital, 10145 Balıkesir, Türkiye; 2 Department of Pulmonology, Faculty of Medicine, Balıkesir University, 10145 Balıkesir, Türkiye; hikmet.coban@balikesir.edu.tr (H.Ç.); fuat.erel@balikesir.edu.tr (F.E.); nurhangencer@hotmail.com (N.S.); mustafa.colak@balikesir.edu.tr (M.C.); merve.senel@balikesir.edu.tr (M.Y.S.); dlvnmnsr@gmail.com (A.D.M.E.); 3 Department of Medical Biochemistry, Faculty of Medicine, Balıkesir University, 10145 Balıkesir, Türkiye; merve.akis@balikesir.edu.tr

**Keywords:** bronchiectasis, hepcidin, inflammation, biomarker, anemia

## Abstract

Hepcidin centrally regulates iron homeostasis and responds to inflammation, iron status, hypoxia, and erythropoietic signaling, yet circulating hepcidin in stable bronchiectasis is poorly characterized. We compared serum hepcidin between 61 patients with stable bronchiectasis and 39 controls without bronchiectasis and assessed associations with anemia, iron metabolism, systemic inflammation, severity, and phenotype. Hepcidin was lower in bronchiectasis (20.34 ± 7.85 vs. 28.34 ± 9.82 ng/mL; median, 20.31 [15.07–25.35] vs. 25.03 [21.00–34.66] ng/mL; *p* < 0.001). It showed no significant associations with anemia status or hematologic, iron metabolism, erythropoietic, or inflammatory indices. Exploratory analyses identified phenotype differences (*p* = 0.002); levels were lowest in chronic obstructive pulmonary disease–bronchiectasis (15.98 ± 6.52 ng/mL), compared with bronchiectasis alone (21.57 ± 6.71) and asthma–bronchiectasis (24.81 ± 9.00). Low hepcidin discriminated bronchiectasis from controls (AUC = 0.729; 95% bootstrap CI: 0.628–0.822); the ≤20.85 ng/mL cutoff yielded 59.0% sensitivity and 79.5% specificity. To our knowledge, this is the first human case–control evidence of reduced circulating hepcidin in stable bronchiectasis. The absence of significant associations with systemic inflammation or anemia/iron status, alongside phenotype differences, suggests that hepcidin may be a candidate biomarker of biological heterogeneity in bronchiectasis not reflected by routine markers.

## 1. Introduction

Bronchiectasis encompasses a heterogeneous group of chronic airway disorders in which permanent bronchial enlargement coexists with defective mucociliary clearance, recurrent infection, and predominantly neutrophilic inflammation [1,2,3]. In a clinically relevant subset, this pathological cycle results in repeated exacerbations, substantial morbidity, and progressive functional decline [1,2]. Although blood-based measures are increasingly being explored to support phenotyping, none has yet become established for routine clinical use [3].

Stable bronchiectasis may be accompanied by measurable systemic inflammation [4], and more recent studies have shown that high-sensitivity C-reactive protein and sputum inflammatory cytokines measured during the stable state may correlate with exacerbation risk and disease severity [5,6]. At the same time, the importance of iron biology in chronic airway diseases is increasingly recognized. Elevated sputum trace metal levels have been reported in inflammatory and suppurative lung diseases, including non-cystic fibrosis bronchiectasis [7], while reviews of nutritional immunity have emphasized that dysregulated pulmonary metal handling represents a biologically plausible but insufficiently investigated pathway in chronic respiratory diseases [8,9].

Because bronchiectasis is a chronic airway disease characterized by marked heterogeneity in etiology, microbiological burden, inflammatory phenotype, radiological extent, and clinical progression, serum and peripheral blood biomarkers are increasingly being investigated as complementary tools for a more objective assessment of disease activity, exacerbation risk, and prognosis [3]. Recent studies of stable bronchiectasis have shown that serum high-sensitivity C-reactive protein levels can predict exacerbation risk over the subsequent year, while larger registry data have demonstrated that stable-state CRP levels are particularly associated with future severe exacerbations [5,10]. Composite indices incorporating C-reactive protein (CRP) with albumin or prealbumin, such as the CRP/albumin ratio and CRP/prealbumin ratio, have been found to be significant predictors of frequent acute exacerbations by jointly reflecting systemic inflammation and nutritional status [11]. Similarly, serum albumin, prealbumin, and fibrinogen levels have been associated with disease severity, hospitalization for respiratory causes, and exacerbation propensity in bronchiectasis; accordingly, these readily accessible parameters have been considered practical biomarker candidates along the inflammation–nutrition–acute-phase response axis [12,13,14,15].

Although they are not strictly serum biomarkers, circulating inflammatory indices derived from routine complete blood counts, such as the neutrophil-to-lymphocyte ratio and the Systemic Immune-Inflammation Index (SII), have also emerged as low-cost markers that may be used to predict disease severity, severe exacerbations, and hospitalization risk in bronchiectasis [16,17,18]. Within the type 2 inflammatory axis, peripheral blood eosinophil counts and serum total IgE have been investigated to identify eosinophilic or T2-high bronchiectasis subgroups; the associations of eosinophilia and eosinopenia with different clinical outcomes indicate that phenotype-dependent biological diversity, rather than a unidirectional inflammatory model, should be considered in bronchiectasis [19,20,21]. Among more specific serum markers, hepatocyte growth factor has been associated with disease severity and future exacerbations; serum desmosine with long-term all-cause and cardiovascular mortality; and serum miR-223-3p with the onset, severe course, and unfavorable prognosis of bronchiectasis [22,23,24]. Taken together, these data suggest that, rather than relying on a single serum biomarker in bronchiectasis, a multimarker approach integrating the acute-phase response, nutritional reserve, cellular inflammation, type 2 responses, tissue remodeling, and molecular regulation offers a more rational strategy for research and clinical phenotyping [3,11,19].

Hepcidin is the principal regulator of systemic iron homeostasis. Although it is synthesized primarily in the liver, hepcidin is also expressed in alveolar macrophages and airway epithelial cells [25,26]. The pulmonary biology of hepcidin is important for host defense because disruption of the hepcidin–ferroportin axis impairs alveolar macrophage function in experimental chronic obstructive pulmonary disease (COPD) [27]. Importantly, hepcidin regulation is not determined solely by inflammation: interleukin-6 (IL-6) induces hepcidin through STAT3 signaling [28], whereas hypoxia and erythropoietic stimulation may suppress hepcidin through the erythropoietin–erythroferrone–BMP/SMAD axis [29,30].

Serum hepcidin patterns in respiratory diseases are heterogeneous. One study reported lower hepcidin levels in patients with more severe or more hypoxemic COPD [31], whereas another study identified higher hepcidin levels and positive correlations with inflammatory markers in stable and exacerbated COPD [32]. Iron-laden airway macrophages have also been associated with recurrent infective COPD exacerbations [33]. In contrast, a recent study of idiopathic pulmonary fibrosis (IPF) reported higher hepcidin levels despite the absence of associations with anemia or routine inflammatory indices [34]. These apparently divergent findings suggest that patterns of hepcidin regulation may be disease-specific in chronic lung diseases.

Despite these observations in other chronic respiratory diseases, serum hepcidin regulation in stable bronchiectasis remains poorly characterized. Bronchiectasis represents a particularly relevant setting because recurrent airway infection, chronic inflammation, hypoxemia in more advanced disease, and common coexisting conditions such as COPD and asthma may influence hepcidin through distinct and potentially opposing pathways. Consequently, it remains unclear whether altered serum hepcidin levels in bronchiectasis reflect the disease itself, conventional determinants such as systemic inflammation, anemia, and iron status, or biological heterogeneity among clinically relevant phenotypes. To address this knowledge gap, the present study compared serum hepcidin levels between patients with stable bronchiectasis and healthy controls and examined their relationships with inflammatory, hematological, iron-related, clinical, and phenotype-related characteristics.

Published data on serum hepcidin in bronchiectasis are limited. We therefore aimed to compare serum hepcidin levels between individuals with bronchiectasis and controls and to evaluate whether hepcidin is associated with routine biomarkers of systemic inflammation or anemia within the bronchiectasis group.

## 2. Materials and Methods

### 2.1. Study Design and Participants

A single-center case–control investigation was carried out between March 2025 and February 2026 in the Department of Chest Diseases at Balıkesir University Health Practice and Research Hospital.

The study included 61 patients with stable bronchiectasis diagnosed according to the criteria of the European Respiratory Society (ERS) 2025 Adult Bronchiectasis Clinical Practice Guideline and 39 controls without bronchiectasis.

The inclusion criteria for patients with bronchiectasis were a diagnosis of bronchiectasis according to the ERS 2025 Adult Bronchiectasis Clinical Practice Guideline and clinical stability. Patients were excluded if they had experienced an acute exacerbation during the preceding three months or had another infectious disease, a rheumatological disorder, hepatic or renal failure, or cancer capable of inducing systemic inflammation. The control cohort consisted of 39 individuals with no bronchiectasis, asthma, or COPD. Comorbidity burden in each group was evaluated using the Charlson Comorbidity Index (CCI).

Age, comorbidities, and other demographic information were documented for participants in both study groups. Pulmonary function testing and modified Medical Research Council (mMRC) dyspnea assessment were performed in both groups at our outpatient clinic. All data were carefully recorded.

For the exploratory phenotype analysis, patients with bronchiectasis and a pulmonologist-confirmed diagnosis of asthma according to GINA criteria were classified into the asthma–bronchiectasis group, whereas those with a diagnosis of COPD according to GOLD criteria were classified into the COPD–bronchiectasis group. Patients without coexisting asthma or COPD were classified as having bronchiectasis alone. Diagnoses were verified through review of the available clinical and spirometric records.

Bronchiectasis cases were additionally stratified into anemic and nonanemic subgroups. Anemia corresponded to hemoglobin < 12 g/dL for women or <13 g/dL for men. The etiologic evaluation consisted of measurements of erythropoietin (EPO), folate, vitamin B12, ferritin, serum iron, and unsaturated iron-binding capacity (UIBC). Calculations were performed for transferrin saturation (TSAT) and total iron-binding capacity (TIBC).

On 25 March 2025, the protocol received approval from Balıkesir University Training and Research Hospital’s Medical Ethics Committee (decision no.: 2025/145). Study conduct followed the Declaration of Helsinki. Written informed consent was obtained from all participants.

### 2.2. Variables and Laboratory Data

Following an overnight fast, blood specimens were obtained from all participants in both cohorts for complete blood count and serum analyses. Samples for serum preparation were placed in yellow-top gel tubes and allowed to clot. Centrifugation was subsequently performed at +4 °C for 10 min at 4000 rpm. A serum portion was transferred to Eppendorf tubes and maintained at −40 °C pending hepcidin analysis.

Complete blood counts were measured on the DxH 800 hematology platform supplied by Beckman Coulter (Brea, CA, USA). The AU680 analyzer from Beckman Coulter (Brea, CA, USA) was used to assay UIBC (unsaturated iron-binding capacity) and serum iron. Quantification of ferritin, vitamin B12, and folate used a UniCel DxI 600 platform supplied by Beckman Coulter (Brea, CA, USA).

Measurement of serum C-reactive protein (CRP) was performed on a BN II platform from Siemens Healthineers (Marburg, Germany). For the erythrocyte sedimentation rate (ESR), an ALS-100 analyzer supplied by Alaris (İzmir, Türkiye) was used.

TIBC was obtained by summing serum iron and UIBC; TSAT (%) was then computed as 100 × serum iron/TIBC. Testing was undertaken in the Biochemistry and Microbiology Laboratory at Balıkesir University Health Practice and Research Hospital.

Gelişim Medical Laboratory measured serum EPO with the Immulite 2000 XPi immunoassay platform (Siemens Healthcare Diagnostics Inc., Flanders, NJ, USA).

#### Serum Hepcidin Analysis

A commercially available enzyme-linked immunosorbent assay was used to quantify serum hepcidin: the Human Hepcidin ELISA kit, catalog no. E-EL-H6202, supplied by Elabscience (Houston, TX, USA). Spectrophotometric readings at 450 nm were acquired on the Varioscan Flash Multimode Reader, a multimode microplate instrument manufactured by Thermo Scientific (Waltham, MA, USA).

Analytical performance of the kit comprised a 0.78–50 ng/mL measurement range, a coefficient of variation below 10%, and a sensitivity of 0.32 ng/mL. Every assay followed the manufacturer’s instructions. Serum hepcidin testing took place in the Medical Biochemistry Laboratory of the Faculty of Medicine at Balıkesir University.

Variables recorded for patients with bronchiectasis included age, Bronchiectasis Severity Index (BSI) score, FACED (F: forced expiratory volume in 1 s [FEV1]; A: age; C: chronic colonization by *Pseudomonas aeruginosa* [PA]; E: radiological extension [number of pulmonary lobes affected]; and D: dyspnea) score, spirometric parameters, modified Medical Research Council (mMRC) dyspnea grade, selected comorbidities, sputum culture data, CRP, white blood cell (WBC) count, hemoglobin, ferritin, serum iron, transferrin saturation, erythropoietin, albumin, fibrinogen, and serum hepcidin.

### 2.3. Statistical Analysis

All computations used SPSS 25.0 (SPSS Inc., Chicago, IL, USA). For descriptive reporting, continuous observations are given as mean ± standard deviation (SD) or median (Q1–Q3), depending on distribution. The Shapiro–Wilk procedure assessed normality. Normally distributed continuous variables are reported as mean ± SD; variables departing from normality are reported as median (Q1–Q3). For independent two-group comparisons, the independent-samples *t*-test was applied to normal data and the Mann–Whitney U test to non-normal data. Counts and percentages [n (%)] were used to summarize categorical variables, which were evaluated by Pearson’s chi-square test. Levene’s test was used to check homogeneity of variance; if equality could not be assumed, a two-group comparison used Welch’s *t*-test. Normally distributed continuous data involving three groups were assessed by one-way analysis of variance (ANOVA). When the omnibus ANOVA was significant, Tukey’s post hoc procedure was applied. Statistical significance was defined by *p* < 0.05, including comparisons between the patient and control cohorts. Relationships of hepcidin with other variables were examined through Spearman’s correlation coefficient. The capacity of serum hepcidin to distinguish bronchiectasis cases from controls was assessed by receiver operating characteristic (ROC) curve analysis. The value yielding the largest Youden index, calculated as sensitivity + specificity − 1, was designated the optimal cutoff. For the area under the curve, the 95% confidence interval was generated through nonparametric bootstrapping with 2000 repetitions.

Multivariable linear regression analyses were performed with serum hepcidin level as the dependent variable and bronchiectasis status (coded as 0 = control and 1 = bronchiectasis) as the primary independent variable. Age and sex were included as prespecified covariates in the primary adjusted model. Hemoglobin and CRP were added in an extended biologically informed sensitivity model, and EPO was further included in an additional model. Covariates were entered concurrently with the entry procedure, and effect estimates are presented as unstandardized B coefficients together with their 95% CIs. Among patients with bronchiectasis, differences in serum hepcidin levels across phenotypes were additionally examined using analysis of covariance, with adjustment for age and sex. Pairwise comparisons of estimated marginal means were adjusted using the Bonferroni method. In a sensitivity analysis, the case group was restricted to patients with bronchiectasis alone, and the between-group difference was estimated after adjustment for age and sex. All tests were two-sided.

## 3. Results

**Figure 1:**
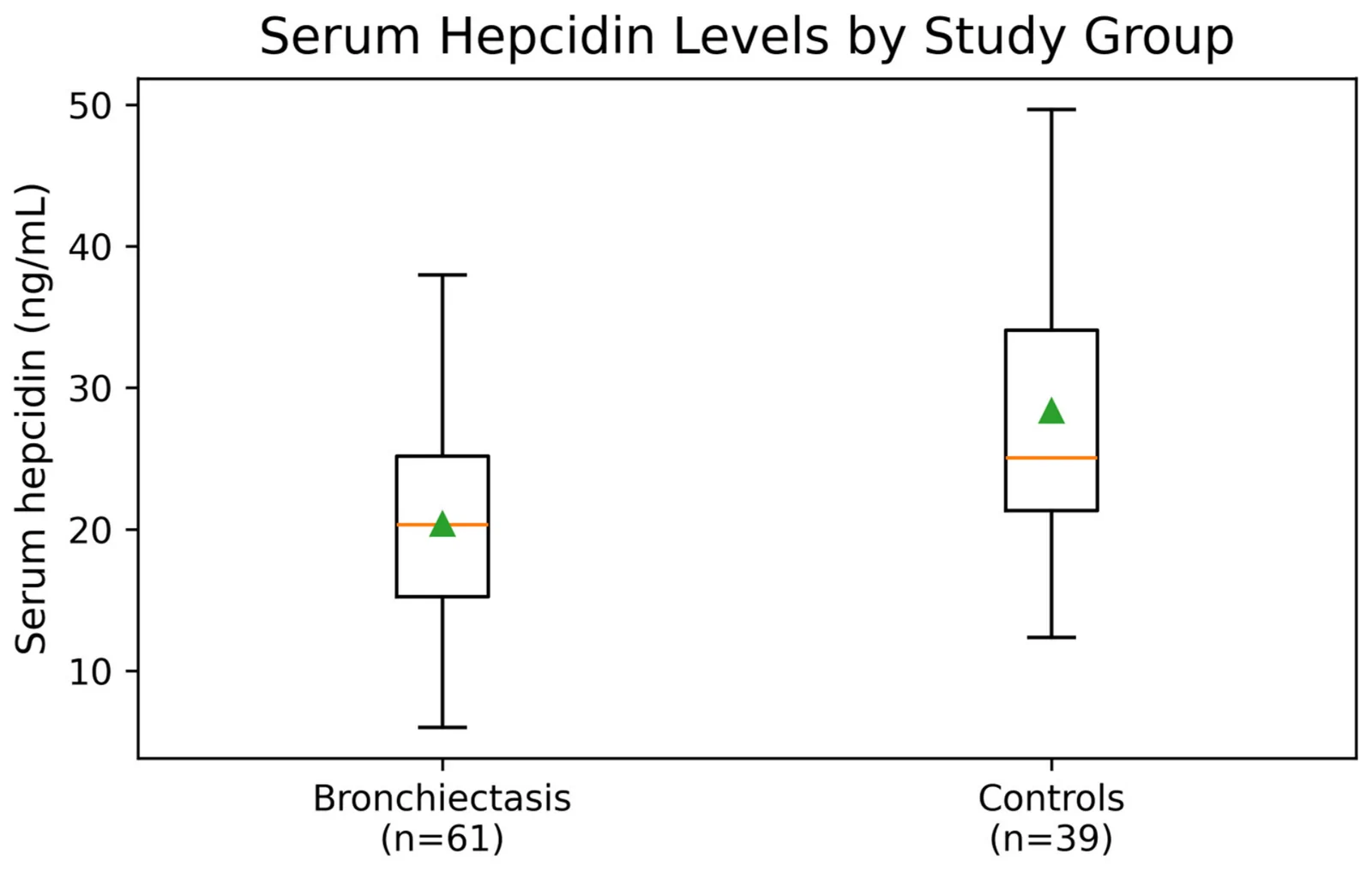
Box plot of serum hepcidin levels in the bronchiectasis (n = 61) and control (n = 39) groups. The boxes represent the interquartile range, the center lines indicate the median, and the triangles indicate the mean.

A total of 61 patients with bronchiectasis and 39 controls were included in the study (Table 1). The patient and control groups were comparable in terms of age, the proportion of women, and the prevalence of sex-specific anemia. Serum hepcidin levels were significantly lower in the bronchiectasis group than in the control group (Table 1; Figure 1). FEV1% predicted and FVC % predicted were lower, whereas the mMRC score was higher, in the bronchiectasis group (Table 1).

In the laboratory comparisons, no significant between-group differences were found in hemoglobin, iron, UIBC (unsaturated iron-binding capacity), TSAT (transferrin saturation), ferritin, folate, vitamin B12, fibrinogen, or the Charlson Comorbidity Index.

In multivariable linear regression analyses, bronchiectasis status remained associated with a lower serum hepcidin level after adjustment for age and sex (B = −7.93 ng/mL; 95% CI, −11.47 to −4.39; *p* < 0.001). The regression coefficient was materially unchanged after further adjustment for hemoglobin and CRP (B = −7.88 ng/mL; 95% CI, −11.49 to −4.27; *p* < 0.001), and the association remained statistically significant when EPO was added to the model (B = −7.65 ng/mL; 95% CI, −11.28 to −4.02; *p* < 0.001).

Based on the definition of Hb < 13 g/dL in men and Hb < 12 g/dL in women, 14 patients with bronchiectasis were anemic, and 47 were non-anemic (Table 2). Hepcidin and EPO levels did not differ between the two groups. Serum iron was numerically lower in anemic patients, but the difference was not statistically significant. No statistically significant differences were observed in ferritin, UIBC, vitamin B12, or folate (Table 2).

No significant correlations were observed between hepcidin and hemoglobin, serum iron, TSAT, ferritin, folate, or erythropoietin (Table 3).

No significant correlations were detected between hepcidin and CRP, ESR, WBC, SII, ferritin, fibrinogen, D-dimer, or albumin (Table 4). Unadjusted serum hepcidin levels differed across phenotype groups and were lowest in the COPD–bronchiectasis group (Table 5). The overall effect of phenotype remained statistically significant after adjustment for age and sex (F(2, 56) = 5.03; *p* = 0.010; partial η^2^ = 0.152). The adjusted estimated marginal means were 21.54 ng/mL (95% CI, 18.71 to 24.38) for the bronchiectasis-alone group, 24.64 ng/mL (95% CI, 20.49 to 28.78) for the asthma–bronchiectasis group, and 16.15 ng/mL (95% CI, 12.71 to 19.59) for the COPD–bronchiectasis group. Pairwise comparisons were interpreted as exploratory. In the unadjusted analysis, Tukey’s post hoc test showed that serum hepcidin levels were significantly lower in the COPD–bronchiectasis group than in the bronchiectasis-alone group (mean difference, −5.59 ng/mL; *p* = 0.026) and the asthma–bronchiectasis group (mean difference, −8.83 ng/mL; *p* = 0.003), whereas the bronchiectasis-alone and asthma–bronchiectasis groups did not differ significantly (*p* = 0.382). Following adjustment for age and sex, Bonferroni-corrected pairwise comparisons indicated that only the difference between the asthma–bronchiectasis and COPD–bronchiectasis groups remained statistically significant (adjusted mean difference, 8.49 ng/mL; adjusted *p* = 0.011); the bronchiectasis-alone group did not differ significantly from either the COPD–bronchiectasis group (adjusted *p* = 0.064) or the asthma–bronchiectasis group (adjusted *p* = 0.653).

Hepcidin levels differed among the BSI groups (Table 5) and were significantly higher in the severe group than in the moderate group (Tukey *p* = 0.005).

Hepcidin levels did not differ among the FACED groups but were significantly higher in patients with at least one hospitalization in the previous year (Table 5).

Hepcidin levels did not differ according to Pseudomonas culture positivity or a history of at least one exacerbation (Table 5). These subgroup analyses were interpreted as exploratory.

In a sensitivity analysis restricted to patients with bronchiectasis alone and controls, serum hepcidin levels remained lower in the bronchiectasis-alone group (21.57 ± 6.71 vs. 28.34 ± 9.82 ng/mL; mean difference, −6.76 ng/mL; 95% CI, −10.83 to −2.70; Welch’s t(63.997) = −3.325; *p* = 0.001). The association remained significant after adjustment for age and sex (B = −6.66 ng/mL; 95% CI, −11.01 to −2.32; *p* = 0.003).

**Figure 2:**
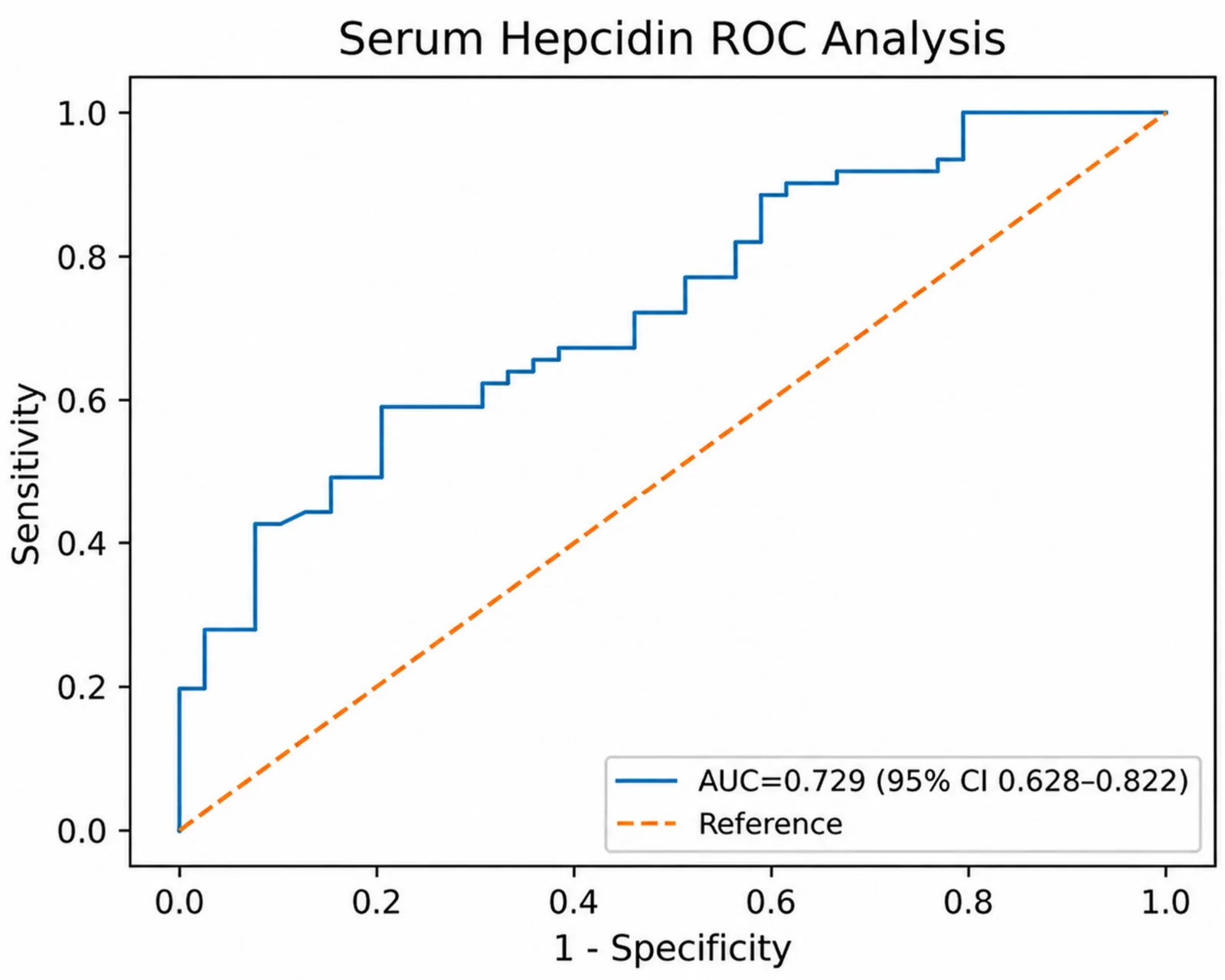
Receiver operating characteristic (ROC) curve for serum hepcidin in discriminating bronchiectasis. Area under the curve (AUC) = 0.729 (95% bootstrap confidence interval (CI) 0.628–0.822); Youden-optimal cutoff ≤20.85 ng/mL, sensitivity 59.0%, specificity 79.5%.

Receiver operating characteristic (ROC) analysis showed that a low serum hepcidin level had modest performance in discriminating bronchiectasis from controls (area under the curve (AUC) = 0.729, 95% bootstrap confidence interval (CI) 0.628–0.822) (Figure 2). The Youden-optimal cutoff for serum hepcidin was ≤20.85 ng/mL; at this cutoff, sensitivity was calculated as 59.0% and specificity as 79.5%.

## 4. Discussion

To the best of our knowledge, this is the first real-world case–control study to compare serum hepcidin levels between patients with stable bronchiectasis and controls. The principal finding of the study was that serum hepcidin levels were lower in patients with bronchiectasis and that this association persisted after adjustment for age and sex. The estimated effect size remained materially unchanged after additional adjustment for hemoglobin, CRP, and EPO. Moreover, the association remained statistically significant in the sensitivity analysis restricted to patients with bronchiectasis alone and controls. Our findings demonstrate that serum hepcidin levels are significantly lower in patients with bronchiectasis and that this reduction cannot be directly explained by anemia, the erythropoietin response, conventional iron metabolism parameters, or the measured markers of systemic inflammation. In the within-patient analyses, the most pronounced reduction in hepcidin was observed in the bronchiectasis phenotype with comorbid COPD, supporting the potential of hepcidin as a distinct candidate serum biomarker reflecting the biological and phenotypic heterogeneity of bronchiectasis. This observation suggests that hepcidin levels may vary across bronchiectasis phenotypes; however, confirmation in larger cohorts is required. Our targeted literature search identified no direct human study comparing serum hepcidin levels between patients with stable non-cystic fibrosis bronchiectasis and healthy controls, suggesting that the present findings address an important biological gap in the bronchiectasis literature.

ROC analysis further supports the potential of serum hepcidin to reflect the biological distinction between the bronchiectasis and control groups. Serum hepcidin demonstrated significant discriminatory performance in distinguishing patients with bronchiectasis from control individuals (AUC = 0.729, 95% bootstrap CI 0.628–0.822). At the cutoff value of ≤20.85 ng/mL determined using the Youden index, sensitivity was 59.0% and specificity was 79.5%, indicating that low hepcidin levels, in particular, may be useful for identifying a bronchiectasis-associated biological phenotype. This finding supports evaluating serum hepcidin as a complementary biomarker that could strengthen the biological characterization of bronchiectasis and aid phenotypic differentiation; this potential warrants validation in future studies. Accordingly, our study positions serum hepcidin as a promising biomarker candidate worthy of further investigation for its potential to reflect phenotype-associated biological differences in bronchiectasis.

The histopathological basis of bronchiectasis is not limited to permanent dilatation of the bronchial lumen; the disease is accompanied by abnormal epithelial repair, disruption of the mucociliary architecture, and chronic inflammatory cell infiltration in both the large and small airways [35]. In a study conducted by Chen et al. using surgically resected lung tissue from 52 patients with non-cystic fibrosis bronchiectasis, epithelial hyperplasia, goblet cell hyperplasia or hypertrophy, and elongated and irregular cilia were demonstrated in both the bronchi and bronchioles [35]. These findings indicate that increased cell numbers in the bronchiectatic airway do not signify preservation of normal epithelial function; rather, cellular hyperplasia may coexist with impaired ciliogenesis, inadequate mucociliary clearance, and loss of functional differentiation [35]. Peng et al. also demonstrated abnormal epithelial and subepithelial proliferation in the peripheral airways of patients with bronchiectasis [36]. Considered together, these two human tissue studies indicate that, rather than “epithelial expansion,” bronchiectasis is more accurately characterized by a hyperplastic but functionally impaired and inadequately differentiated distal airway epithelium [35,36]. In our study, serum hepcidin levels were lower in the bronchiectasis group than in the control group. Airway epithelial alterations may be relevant to local hepcidin biology in bronchiectasis, although their relationship with circulating hepcidin remains to be established.

Hepcidin is one of the principal regulators of systemic iron homeostasis, and hepatocytes are the main source of circulating hepcidin [37]. Nevertheless, hepcidin production is not confined to the liver. Frazier et al. demonstrated that differentiated human bronchial epithelial cells express hepcidin and that the presence of hepcidin in the airways does not alter cellular iron transport [26]. These findings support the view that airway epithelium-derived hepcidin may contribute to local antimicrobial defense or paracrine intercellular communication rather than to systemic iron metabolism [26]. Alveolar macrophages have also been shown to express hepcidin and to increase its expression in response to bacterial lipopolysaccharide stimulation, suggesting the presence of a locally regulated network involving the epithelium, macrophages, and iron metabolism in the lung [25]. These experimental observations concern locally produced hepcidin, whereas our study assessed circulating hepcidin, which was not significantly correlated with parameters of iron metabolism or anemia in patients with stable bronchiectasis.

Serum hepcidin has been investigated across several clinical conditions and biomarker applications involving systemic inflammation or altered iron regulation, including Kawasaki disease, severe acute pancreatitis, the detection of autologous blood transfusion, chronic kidney disease, inflammatory bowel disease, Behçet’s disease, and obstructive sleep apnea [38,39,40,41,42,43,44,45]. Collectively, these studies demonstrate the broader clinical interest in hepcidin as a potential marker related to inflammation and iron homeostasis, although its behavior and clinical relevance may vary across diseases. This background provided the rationale for investigating serum hepcidin in bronchiectasis, for which evidence remains limited.

One study reported that CRP levels in patients with stable bronchiectasis were positively correlated with both BSI and FACED scores (r = 0.30; *p* = 0.001 for both scores) [46]. Similarly, a more recent study reported positive correlations between CRP levels and BSI and E-FACED scores (r = 0.28 and r = 0.24, respectively) [47]. These findings support the possibility that the systemic inflammatory burden in stable bronchiectasis may be associated with disease severity. However, in our study, CRP levels did not differ between the bronchiectasis and control groups (*p* = 0.132); moreover, no significant correlations were observed between serum hepcidin levels and CRP, erythrocyte sedimentation rate, SII, or ferritin. These results suggest that the observed reduction in hepcidin may not be directly explained by the measured inflammation-related parameters.

Within this biological framework, persistent infection, neutrophilic inflammation, protease activity, and recurrent epithelial injury in bronchiectasis may promote the development of a hyperplastic but dysfunctional epithelial phenotype in place of normally differentiated ciliated and secretory epithelium, thereby reducing local hepcidin antimicrobial peptide (*HAMP*) expression or the hepcidin response to appropriate stimuli. This process may be better explained by a multicomponent model involving both bronchiectasis-associated pulmonary epithelial remodeling and systemic regulators of hepcidin [35,48].

From the perspective of this mechanistic model, the lower hepcidin levels in patients with bronchiectasis and comorbid COPD than in those with other bronchiectasis phenotypes constitute the most noteworthy subgroup result of our study (Table 5). Regarding studies of hepcidin in COPD, Duru et al. reported lower serum hepcidin levels in patients with moderate and severe stable COPD than in healthy controls [31]. In contrast, Tandara et al. reported increased serum hepcidin levels in patients with COPD during both stable disease and exacerbations and found this increase to be associated with IL-6 and systemic inflammation [32]. A more recent study reported that the levels of different hepcidin isoforms were similar in patients with COPD and the general population, that these isoforms were associated with CRP and ferritin, and that the initiation of long-term oxygen therapy did not produce the anticipated marked increase in hepcidin levels [49]. These divergent results indicate that circulating hepcidin in COPD does not reflect a single, fixed feature of the disease; rather, the net result is jointly determined by IL-6 signaling that increases systemic inflammation, hepcidin-suppressive influences such as hypoxia and erythropoietic activity, iron status, disease stage, and the assay method used [31,32,49].

Because circulating hepcidin is produced predominantly by hepatocytes, reduced *HAMP* expression in the airway epithelium, although potentially contributing indirectly, is unlikely on its own to quantitatively account for the lower serum hepcidin concentrations observed in our cohort. Epithelial dysfunction may instead represent a concomitant local manifestation of pulmonary remodeling, whereas serum hepcidin likely reflects the hepatic integration of opposing systemic signals. IL-6 signaling upregulates hepatic *HAMP* expression during inflammation [28], whereas hypoxia-driven erythropoiesis acts in the opposite direction. In healthy volunteers exposed to high altitude, an early increase in erythropoietin (EPO) was followed by a marked decrease in serum hepcidin [50]. Erythroferrone (ERFE), secreted by erythroblasts, has been shown to mediate the suppression of hepatic hepcidin, thereby increasing iron availability for erythropoiesis [30]. Furthermore, a human experimental endotoxemia model demonstrated that hypoxia increased EPO and ERFE levels and attenuated the inflammation-induced rise in hepcidin, supporting the ability of hypoxic–erythropoietic signals to counterbalance inflammatory induction [51]. This systemic framework may be particularly relevant to the COPD–bronchiectasis phenotype: lower serum hepcidin levels have been reported in patients with stable moderate or severe COPD, with hepcidin showing a positive association with oxygenation [31]. Consistently, in a murine COPD model exposed to cigarette smoke, hepatic *HAMP* expression and circulating hepcidin concentrations decreased, whereas EPO and bone marrow ERFE increased [27]. Accordingly, intermittent or subclinical hypoxemia, smoking-related erythropoietic drive, and increased systemic iron demand may contribute to lower serum hepcidin levels, particularly in the COPD–bronchiectasis phenotype; however, this does not imply that airway-derived hepcidin is a major source of the circulating peptide. The absence of correlations with routine hematologic, iron metabolism, and inflammatory indices does not exclude this model, because single-time-point peripheral measurements may fail to capture intermittent changes in oxygenation or bone marrow-to-liver signaling. Therefore, local epithelial dysfunction should be regarded as a component of pulmonary hepcidin dysregulation but not as the sole or direct explanation for the reduction in serum hepcidin. This integrated lung–systemic model warrants further investigation through the simultaneous assessment of oxygenation, IL-6, ERFE, hepatic *HAMP* expression, and related regulatory pathways.

In another study, mortality rates were higher among patients with bronchiectasis and comorbid COPD than among those without COPD. Patients with bronchiectasis and COPD experienced more frequent exacerbations, exacerbation-related hospitalizations, and intensive care unit admissions during the previous year than those without COPD [52]. Evidence of exhaustion and impaired regenerative capacity in airway basal progenitor cells in both early and established COPD also indicates that normal epithelial renewal is compromised [53]. Thus, the COPD + bronchiectasis phenotype may represent a more extensive pattern of epithelial dysfunction in which COPD-related barrier impairment, progenitor cell depletion, and alveolar injury are superimposed on bronchiectasis-associated abnormal bronchial remodeling [35,53,54].

Our finding of low hepcidin in bronchiectasis differs in direction from the increase in hepcidin reported in idiopathic pulmonary fibrosis; in the IPF study, serum hepcidin levels were higher than those in controls, although hepcidin was not correlated with anemia or the measured parameters of systemic inflammation [34]. The finding of high hepcidin in IPF, low hepcidin in bronchiectasis, and even lower hepcidin in the COPD + bronchiectasis phenotype suggests that the hepcidin response does not follow the same direction across all chronic lung diseases and that fibrotic alveolar remodeling and destructive airway and emphysematous remodeling phenotypes may produce different hepcidin profiles [31,34].

Although FACED and BSI scores are correlated, BSI has been reported to classify patients into higher severity categories than FACED [55]. In our study, although hepcidin levels differed among BSI categories, the absence of a linear relationship and the lack of differences among FACED categories may have been partly attributable to differences in the variables included in the two scores and their respective weightings. Therefore, these findings suggest that hepcidin may be a phenotype-associated biomarker capable of reflecting the biological heterogeneity of bronchiectasis and airway–parenchyma–macrophage interactions.

The apparently discordant increase in hepcidin in the severe BSI and previously hospitalized subgroups may reflect a competing inflammatory signal rather than a reversal of the overall case–control finding. Because previous hospitalization is a heavily weighted component of the BSI, these observations partly overlap rather than represent independent associations [56]. Severe exacerbations requiring hospitalization elicit a stronger systemic inflammatory response, with IL-6 and hsCRP elevations persisting to day 30 [57]; because IL-6 directly induces hepatocytic *HAMP* expression through STAT3 [28], recurrent inflammatory activation could partially offset hepcidin-lowering influences, producing the observed nonlinear pattern and values that numerically approached the control mean without establishing normalization. A single stable-state CRP measurement may not capture such intermittent exposure; nevertheless, these exploratory subgroup findings should be interpreted cautiously.

Overall, human tissue and cell-culture studies support local epithelial dysregulation of hepcidin, whereas the serum findings point to a parallel systemic regulatory process. The pronounced reduction in serum hepcidin observed in the COPD–bronchiectasis group is compatible with a two-compartment model in which pulmonary epithelial dysfunction coexists with hepatic hepcidin-suppressive signaling; however, it does not establish that reduced airway *HAMP* expression caused the decrease in circulating hepcidin. In future studies, this model could be tested by simultaneously assessing serum hepcidin; hepcidin in bronchoalveolar lavage fluid or sputum; *HAMP* expression in bronchial biopsy specimens; ferroportin in alveolar macrophages; and oxygenation, IL-6, EPO, and ERFE. Such an approach could help distinguish local pulmonary dysregulation from systemic hepatic regulation of circulating hepcidin and clarify the biological basis of the observed phenotypic association. Overall, these results support further evaluation of serum hepcidin as a candidate marker of biological heterogeneity in bronchiectasis in larger, independent cohorts.

### Study Limitations

The single-center setting and the small numbers within the phenotype and anemia subgroups should be considered when interpreting the findings. These subgroup sizes may have limited statistical power and reduced the certainty of the exploratory comparisons. Moreover, the analysis was confined to serum hepcidin; hepcidin in sputum, bronchoalveolar lavage fluid, or bronchial biopsy specimens, together with alveolar macrophage ferroportin, was not assessed. Larger multicenter cohorts are required to confirm the present observations.

## 5. Conclusions

Serum hepcidin levels were markedly lower in patients with bronchiectasis than in controls without bronchiectasis. The lack of significant associations between hepcidin and parameters of anemia, iron metabolism, or systemic inflammation suggests that the observed change may reflect a bronchiectasis-specific biological process distinct from these mechanisms. The pronounced reduction in hepcidin in bronchiectasis with comorbid COPD, together with the significant differences among BSI groups, supports hepcidin as a potential indicator of the clinical heterogeneity of bronchiectasis. Our findings identify serum hepcidin as a distinct and promising candidate biomarker for the biological characterization and phenotypic differentiation of bronchiectasis.

## Figures and Tables

**Table 1:** Comparison of the demographic, clinical, and laboratory characteristics of the patient and control groups.

Variable	Patient Group	Control Group	*p* Value
Age, years	55.00 (47.50–70.00)	65.00 (40.00–75.00)	0.671
Female sex	29 (47.5%)	22 (56.4%)	0.387
Anemia (sex-specific Hb definition)	14 (23.0%)	7 (17.9%)	0.549
Hepcidin, ng/mL	20.31 (15.07–25.35)	25.03 (21.00–34.66)	<0.001
FEV1/FVC, %	79.00 (72.00–84.50)	77.00 (71.00–85.00)	0.508
FEV1, % predicted	65.00 (53.00–83.50)	83.00 (72.00–101.00)	<0.001
FVC, % predicted	67.34 ± 18.81	90.00 ± 18.03	<0.001
mMRC score	1.00 (1.00–2.00)	0.00 (0.00–1.00)	<0.001
Erythropoietin, mIU/mL	10.60 (7.67–18.68)	10.40 (5.63–15.00)	0.329
ESR, mm/h	12.00 (7.00–18.00)	11.00 (7.00–18.00)	0.673
CRP, mg/L	3.06 (3.06–3.49)	3.00 (2.00–4.54)	0.132
WBC, ×10^9^/L	7.50 (5.90–8.50)	7.80 (6.60–8.50)	0.382
Hemoglobin, g/dL	13.20 (12.70–14.45)	13.40 (12.80–13.50)	0.311
Neutrophil count, ×10^9^/L	4.30 (3.50–5.40)	4.80 (4.10–5.30)	0.249
Lymphocyte count, ×10^9^/L	1.93 ± 0.71	2.02 ± 0.62	0.485
Serum iron, µg/dL	64.00 (42.50–87.00)	61.50 (44.25–77.50)	0.379
UIBC, µg/dL	277.00 (235.50–327.00)	298.00 (241.00–347.00)	0.519
TSAT, %	19.13 (11.76–26.11)	18.02 (11.92–29.47)	0.867
Ferritin, µg/L	33.00 (10.65–66.85)	26.70 (10.98–82.95)	0.701
Fibrinogen, mg/dL	319.00 (287.00–359.00)	336.00 (273.75–406.00)	0.205
Folate, µg/L	9.10 (6.95–12.80)	9.80 (6.20–13.90)	0.896
Vitamin B12, ng/L	239.00 (176.00–463.50)	211.00 (136.75–334.50)	0.115
SII	575.80 (378.79–786.70)	713.45 (454.57–856.00)	0.344
Charlson Comorbidity Index	3.00 (3.00–4.00)	3.00 (3.00–4.00)	0.879

Notes: Continuous variables are presented as mean ± SD or median (Q1–Q3), according to normality, and categorical variables as n (%). *p* values were calculated using the independent-samples *t*-test (FVC and lymphocyte count), Mann–Whitney U test (other continuous variables), or Pearson’s chi-square test (categorical variables). Anemia: Hb < 13 g/dL in men and Hb < 12 g/dL in women. Abbreviations: CRP, C-reactive protein; ESR, erythrocyte sedimentation rate; FEV1, forced expiratory volume in 1 s; FVC, forced vital capacity; mMRC, modified Medical Research Council; SD, standard deviation; SII, Systemic Immune-Inflammation Index; TSAT, transferrin saturation; UIBC, unsaturated iron-binding capacity; WBC, white blood cell count.

**Table 2:** Comparison of hepcidin and anemia-related parameters according to sex-specific anemia status in the bronchiectasis patient group.

Variable	Anemic	Non-Anemic	*p* Value
Age, years	53.00 (50.50–72.00)	56.00 (45.00–70.00)	0.803
Hepcidin, ng/mL	18.86 ± 6.08	20.78 ± 8.31	0.426
Ferritin, µg/L	17.65 (10.03–54.15)	39.80 (12.80–67.20)	0.186
Hemoglobin, g/dL	11.85 ± 0.96	13.97 ± 1.18	<0.001
Serum iron, µg/dL	56.00 (32.00–68.50)	74.00 (46.00–92.00)	0.080
UIBC, µg/dL	319.93 ± 83.42	279.17 ± 55.20	0.104
Erythropoietin, mIU/mL	12.00 (3.50–20.80)	10.30 (7.95–15.55)	0.881
Vitamin B12, ng/L	231.00 (154.50–608.00)	266.00 (178.00–409.00)	0.764
Folate, µg/L	9.75 (6.80–13.30)	9.00 (6.90–12.60)	0.699

Notes: Continuous variables are presented as mean ± SD or median (Q1–Q3), according to normality. *p* values were calculated using the independent-samples *t*-test (hepcidin and hemoglobin), Welch’s *t*-test (UIBC), or Mann–Whitney U test (other variables). Abbreviations: SD, standard deviation; UIBC, unsaturated iron-binding capacity.

**Table 3:** Correlations between hepcidin and anemia, erythropoiesis, and iron metabolism parameters in patients with bronchiectasis.

Variable	Spearman r	*p* Value
UIBC, µg/dL *	−0.168	0.197
Vitamin B12, ng/L	−0.036	0.784
Serum iron, µg/dL	0.083	0.527
Ferritin, µg/L	−0.074	0.569
Folate, µg/L	−0.079	0.547
Hemoglobin, g/dL	−0.016	0.905
TSAT, %	0.153	0.238
Erythropoietin, mIU/mL	−0.221	0.096

Notes: Spearman’s rank correlation was used. * UIBC, unsaturated iron-binding capacity; TSAT, transferrin saturation.

**Table 4:** Correlations between hepcidin and inflammatory parameters in the bronchiectasis patient group.

Variable	Spearman r	*p* Value
SII *	−0.144	0.272
Ferritin, µg/L	−0.074	0.569
CRP, mg/L	0.086	0.511
ESR, mm/h	−0.041	0.756
WBC, ×10^9^/L	0.083	0.524
Fibrinogen, mg/dL	−0.095	0.468
D-dimer, mg/L	−0.006	0.964
Albumin, g/L	−0.034	0.794

* SII: Systemic Immune-Inflammation Index. CRP: C-reactive protein; ESR: erythrocyte sedimentation rate; WBC: white blood cell count.

**Table 5:** Hepcidin levels by phenotype, disease severity, and clinical subgroup in patients with bronchiectasis.

Grouping	n	Category	Hepcidin, ng/mL	*p* Value
Bronchiectasis phenotype	27	Bronchiectasis alone	21.57 ± 6.71	0.002
	13	Asthma–bronchiectasis	24.81 ± 9.00	
	21	COPD–bronchiectasis	15.98 ± 6.52	
BSI severity	30	Mild	19.84 ± 6.78	0.006
	16	Moderate	16.61 ± 8.51	
	15	Severe	25.30 ± 6.94	
FACED severity	28	Mild	19.76 ± 6.78	0.807
	19	Moderate	20.35 ± 8.11	
	14	Severe	21.47 ± 9.77	
Pseudomonas culture	51	Negative	20.18 ± 8.12	0.728
	10	Positive	21.13 ± 6.57	
≥1 exacerbation in the past 1 year	37	No	19.36 ± 6.65	0.265
	24	Yes	21.85 ± 9.35	
≥1 hospitalization in the past 1 year	45	No	18.78 ± 7.44	0.008
	16	Yes	24.71 ± 7.51	

Notes: Data are presented as mean ± SD. *p* values were calculated using one-way ANOVA (phenotype, BSI, and FACED groups), independent-samples *t*-test (Pseudomonas culture and hospitalization), or Welch’s *t*-test (exacerbation history). Abbreviations: BSI, Bronchiectasis Severity Index; COPD, chronic obstructive pulmonary disease; FACED, forced expiratory volume in 1 s, age, chronic Pseudomonas aeruginosa colonization, radiological extension, and dyspnea.

## Data Availability

The data presented in this study are available on request from the corresponding author due to privacy and ethical restrictions.
